# Spectroscopic and Molecular Methods to Differentiate Gender in Immature Date Palm (*Phoenix dactylifera* L.)

**DOI:** 10.3390/plants10030536

**Published:** 2021-03-12

**Authors:** Abdul Latif Khan, Ahmed Al-Harrasi, Muhammad Numan, Noor Mazin AbdulKareem, Fazal Mabood, Ahmed Al-Rawahi

**Affiliations:** 1 Natural & Medical Sciences Research Center, University of Nizwa, Nizwa 616, Oman; abdullatif@unizwa.edu.om (A.L.K.); noor@unizwa.edu.om (N.M.A.); ahmed@unizwa.edu.om (A.A.-R.); 2 Department of Biology, University of North Carolina, Greensboro, NC 27402-6170, USA; m_numan@uncg.edu; 3 Institute of Chemical Sciences, University of Swat, Khyber Pakhtunkhwa 19200, Pakistan

**Keywords:** *Phoenix dactylifera*, NIR and FTIR spectroscopy, PLS regression, NMR, chemometrics, RT-PCR, transcript accumulation, sex

## Abstract

*Phoenix dactylifera* (date palm) is a well-known nutritious and economically important fruit tree found in arid regions of the Middle East and North Africa. Being diploid, it has extremely high divergence in gender, where sex differentiation in immature date palms (*Phoenix dactylifera* L.) has remained an enigma in recent years. Herein, new robust infrared (near-infrared reflectance spectroscopy (NIRS) and Fourier transform infrared attenuated total reflectance (FTIR/ATR)) and nuclear magnetic resonance (NMR) spectroscopy methods coupled with extensive chemometric analysis were used to identify the sex differentiation in immature date palm leaves. NIRS/FTIR reflectance and ^1^H-NMR profiling suggested that the signals of monosaccharides (glucose and fructose) and/or disaccharides (maltose and sucrose) play key roles in sex differentiation. The three kinds of spectroscopic data were clearly differentiated among known and unknown male and female leaves via principal component and partial least square discriminant analyses. Furthermore, sex-specific genes and molecular markers obtained from the lower halves of LG12 chromosomes showed enhanced transcript accumulation of mPdIRDP52, mPdIRDP50, and PDK101 in females compared with in males. The phylogeny showed that the mPdIRD033, mPdIRD031, and mPdCIR032 markers formed distinctive clades with more than 70% similarity in gender differentiation. The three robust analyses provide an alternative tool to differentiate sex in date palm trees, which offers a solution to the long-standing challenge of dioecism and could enhance in situ tree propagation programs.

## 1. Introduction

Horticultural crops and fruit-bearing trees are economically and nutritionally important to humans; however, identification and propagation of their reproductive stage is also essential to ensure improved plant production. Male- and female-related genes are often closely linked and are located on one chromosome [1,2]. Chromosomes related to sex differentiation evolve by non-recombinant location across the sex-related genes, causing instance translocations, duplications, inversions, and deletions [3,4]. The distribution of heterogametic males quite often prevails in dioecious plant species [5]. Some previous studies of dioecious plants have shown close links between molecular markers, and their analysis shows essential information about the occurrence of the XY-chromosome system. This was also revealed in *Dioscorea tokoro* [6], *Carica papaya* [7], and Asparagus [8].

*Phoenix dactylifera* (Arecaceae) is a diploid (2n; 2x-36) that has an extremely high divergence in sex at the flowering level. The floral bud specifically becomes bisexual with similar male and female primordia, which usually go through selective abortion in the flowers [9]. Since ancient times, the date palm has been known as one of the most nutritious and economically important trees of the arid regions, for example, the Middle East and North Africa. It is also regarded as one of the major crops in the desert land ecosystem [10]. A date palm tree is usually propagated through vegetative offshoots or tissue-culture-based approaches to maintain the specific properties of the fruit [11]. Currently, the genetic diversity of date palm trees and their breeding methodologies are being threatened by exposure to a wide array of environmental and biological pressures. This has led to investment in highly tolerant varieties through intensive breeding programs [12,13]. However, such programs are time-consuming, not only in terms of efficient plant growth, but also in the selection and differentiation of male and female germplasms for further cultivation and propagation.

This is the reason why it is of utmost importance to consider methods and approaches to determine the sex in date palms at an immature stage. Identifying female plants, which are responsible for fruit production, certainly enhances the uniformity in crossing and marker-assisted selection programs. Sex differentiating molecular markers have also been designed for date palms [14]. Al-Mahmoud et al. [15] showed molecular markers related to the identification of males in date palm samples; however, such markers are difficult to use in extensive programs with lower sex-discrimination percentages. The sex-related progeny has been shown to be half males, which reveals that in date palm, sex is often determined through a single chromosome locus [16,17,18,19]. However, the currently used sex differentiation processes and approaches are uncertain with low reliability for determining whether a date palm is male or female before its final reproductive stage [20]. Several molecular methods reported so far have advantages and disadvantages and suffer from robustness. Therefore, the development of simple and reliable methods is mandatory to allow broader date palm tree propagation and commercialization.

There are also analytical and molecular approaches that can be used to explain the distinction between sexes [21,22], each having advantages and disadvantages. There have been no previous reports on the discrimination between sexes in date palm trees through vibrational and nuclear magnetic resonance spectroscopy. NIRS can measure the chemical structure and composition of a sample based on the absorption of near-infrared radiation by CO–H and N–H bonds, resulting in overtones and variations in bands or peaks that can be observed at wavelengths of 780–2500 nm [23,24]. Being a robust tool, there are few examples of the use of NIRS to carry out phenotypic, metabolomic, and physiological assessments of trees [25]. These analysis methods, coupled with the use of molecular methods, such as the polymerase chain reaction (PCR) and real-time (RT)-PCR are widely used to determine the expression and distribution of markers and genes in plants. We could not find any credible protocol involving the partial least square discriminant analysis (PLS-DA) that models sex-specific distinction on date palms in the literature. Thus, the current study aimed to understand sex differentiation in immature leaf samples of male and female date palms using spectroscopy, regression, NMR, and molecular techniques. 

## 2. Results and Discussion

### 2.1. NIRS Based Discrimination in Sex of Date Palm

**Figure 1:**
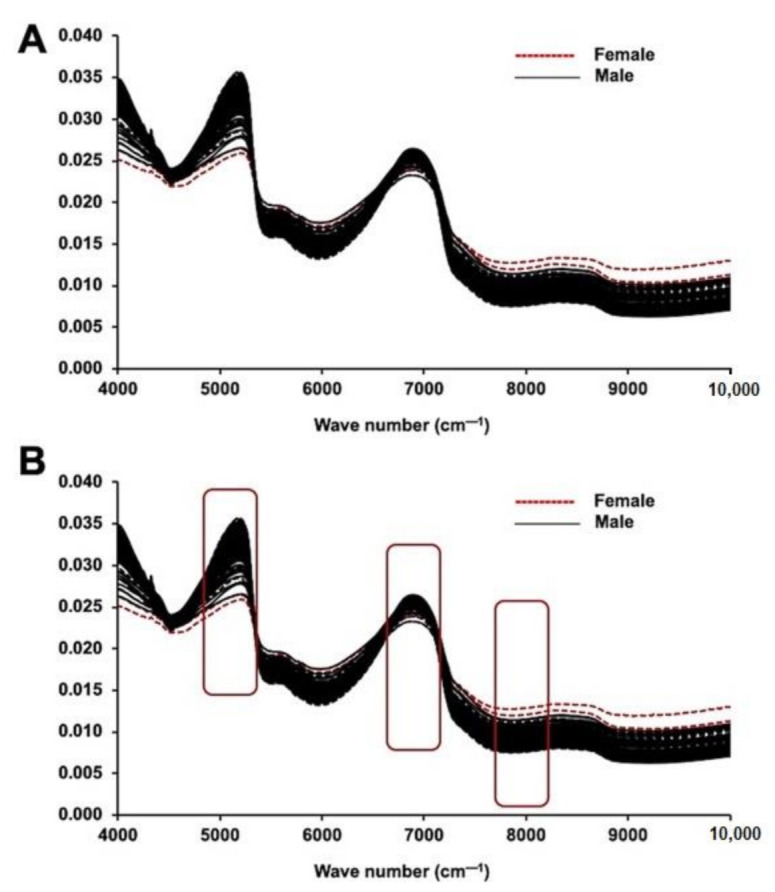
NIRS analysis of immature date palm leaf samples. (**A**) Raw NIR spectra (without pre-processing) and (**B**) pre-processed NIR spectra (Unit vector Normalization) of both male and female immature date palm leaf samples. The figure demonstrates the scattering effect in the wavenumber range of 4000 to 10,000 cm^−1^ due to absorbance without preprocessing. The spectra represent the fifty individual samples each from male and female and that were tested five times.

The NIRS spectral data for male and female plants were assessed for possible points of discrimination between sexes (Figure 1A,B). Absorption peaks in NIRS spectra occurred because of the combination and overtone of vibrations/absorptions by C–H hydrocarbons in the sample. Absorption peaks (Figure 1A,B; Figure S1) in NIRS spectral data were found in the 3500 to 3700 cm^−1^ regions, which suggests an –OH composition. Additionally, the peaks in the 7100 to 7700 cm^−1^ regions were attributed to the absorption of 1st overtones in the ethanol –OH group. The peaks from 8264 to 8800 cm^−1^ are predicted to be the result of secondary overtones of C–H from the –CH_3_ group at 8264 cm^−1^ and the aromaticity of C–H at 8726 cm^−1^ in the male and female leaf samples. Unit vector normalization pre-processing was performed at the optimum level to reduce the scattering effects in the samples. A few studies have reported the effectiveness of NIRS for classifying samples such as gourd seeds [2], seed lots of agricultural crops [26], spinach seeds [27], and soybean seeds [28], suggesting the potential use and validity of this method for biological samples. The current analysis also showed delicate variation in male and female date palm samples.

**Figure 2:**
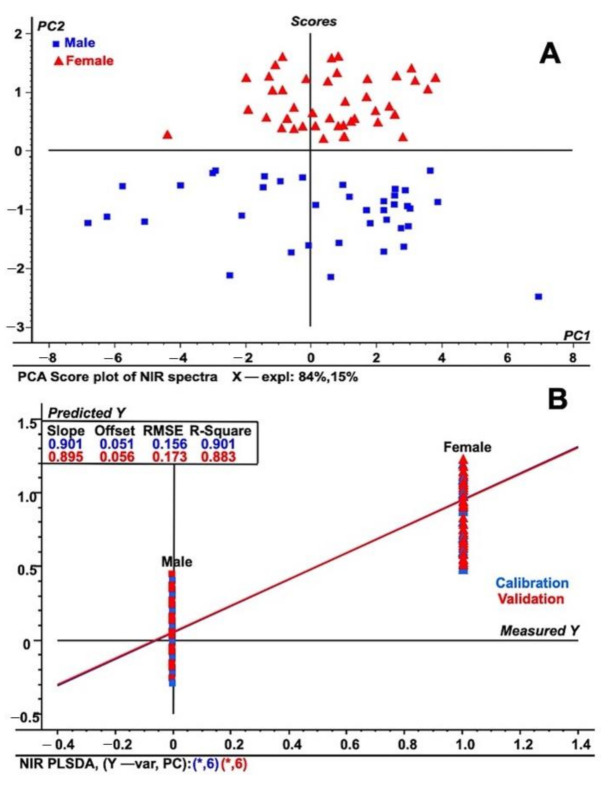
Regression analysis used for immature date palm leaf samples (**A**) PCA score plot of NIR spectral data for both male and female leaf samples from immature date palms. (**B**) PLS-DA plot using NIR spectral data from both male and female date palm leaf samples. The figure shows both the calibration and validation of the tested samples using spectral data in the wavenumber range of 4000 to 10,000 cm^−1^. The spectra represent the fifty individual samples each from male and female and that were tested five times.

To further differentiate the samples into males and females, detailed regression and exploratory methods were performed. The principal component analysis (PCA) exploratory data analysis tool was applied to unit vector normalized NIR spectral data [29] to differentiate among male and female date leaf samples (Figure 2A). The PCA analysis provided additional validation in the assessment of how these two males and female samples were differentiated and was used to form separate groups (Figure 2A). The groups identified were located in different parts of the score plot of the PCA model, allowing complete differentiation of male and female sexes from one other. The differentiation was based on the differences in respective NIRS spectra due to chemical changes in the C–H–O bonding patterns. Figure 2A also shows that 84% of the total NIR spectral variation in PC1 was used to build the PCA score plot, while PC2 explained 15% of the spectral variation. The current approach was also validated by a recent study on NIRS that helped to distinguish between viable and non-viable tree seeds [30]. In comparison, experiments suggested the use of NIRS to solve problems related to ecological analysis and modeling, for example, the identification of young and mature Amazonian tree species [31]. Furthermore, utilizing NIR with multivariate modeling methods, such as PLS (partial least squares) regression, contributed to the solving of many questions related to the growth and dynamics of specific ecosystems [32,33,34,35,36,37]. A study suggested that the current PLS regression model is a more accurate method for use in ecological modeling [37,38]. Following similar statistical approaches, Ohsowski utilized the PLS regression model to accurately assess above-ground plant biomass using multiple collinear plant traits to generate a standard curve [39].

In addition, previous reports have shown that the PLS-DA model suggests a high degree of prediction and discrimination among plant samples of the same kind [34,35,36,37,38,39]. Consequently, we developed and also applied a similar model to differentiate between male and female date leaf parts based on the obtained normalized NIRS spectral data, as shown in Figure 2B. As shown in Figure 2B, male date palms fall in the lower part of PLS-DA model based on the NIRS spectral data. Additionally, further discrimination of male and female date leaf samples was performed through PCA analysis of the PLS-DA model (Figure S3). The score plot shows that female leaf samples are segregated and isolated from male ones on one side of the PLS-DA score plot. There are various examples in the recent literature where the authors have shown clear demarcation between sexes across a wide array of samples [32,33,34,35,36,37]. To further investigate sex differentiation in date palms, we used FT-IR/ATR coupled with measurement of the reflected spectral information on both kinds of leaf in the wavelength range of 4000 to 400 cm^−1^ (see preceding section).

### 2.2. FT-IR Based Discrimination Date Palm Sex

**Figure 3:**
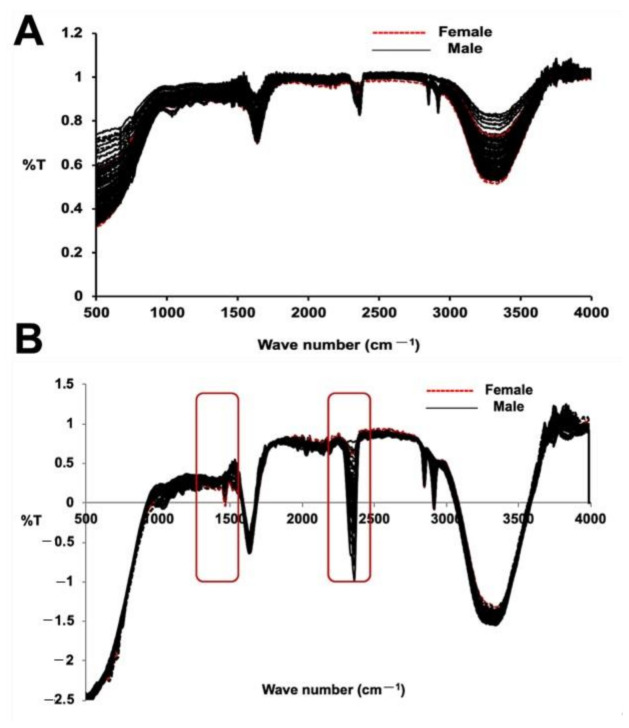
FTIR/ATR analysis for immature date palm leaf samples (**A**) FTIR (ATR) spectra (without pre-processing) and (**B**) pre-processed male and female immature date palm leaf samples. The figure shows the scattering effect due to absorbance without preprocessing in the wavelength range of 400 to 4000 cm^−1^. The spectra represent the fifty individual samples each from male and female and that were tested five times. (Unit vector normalization, SNV, S. Golay 10 smoothing point pre-processing).

**Figure 4:**
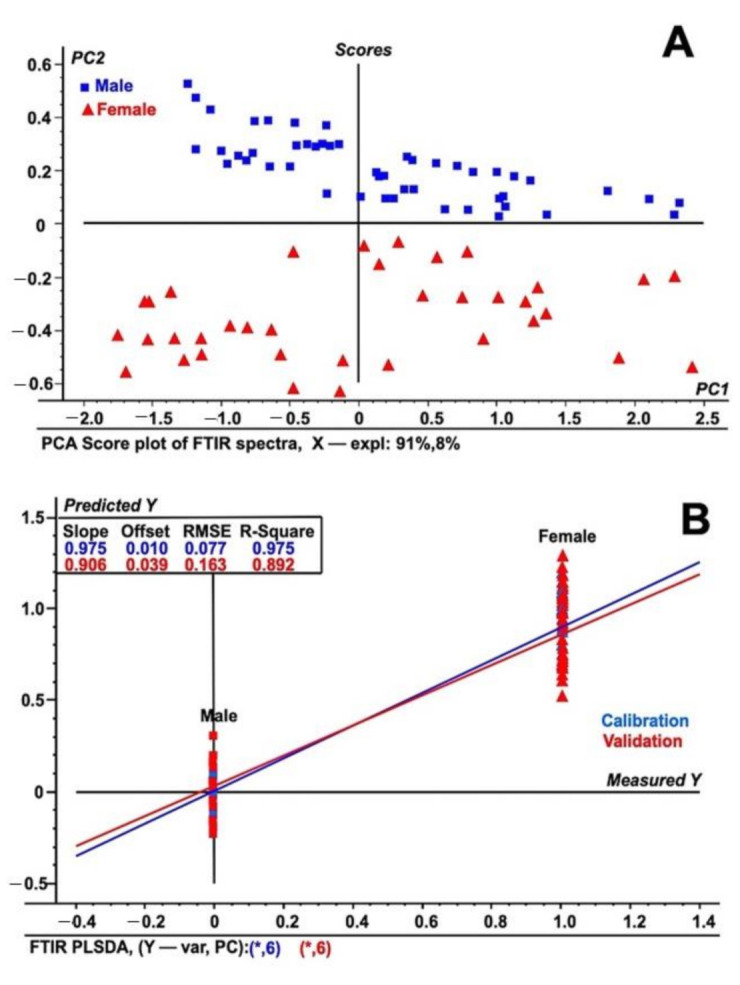
PLSDA analysis of immature leaf samples of Date palm (**A**) PCA score plot using FTIR ATR spectral data from male and female date palm leaves samples. (**B**) PLS-DA plot using FTIR ATR spectral data from both male and female date palm leaf samples. The figure shows both the calibration and validation of the tested samples using spectral data in the wavelength range of 400 to 4000 cm^−1^. The spectra represent the fifty individual samples each from male and female and that were tested five times.

The FT-IR spectral data for both the male and female date palm leaf samples are illustrated in Figure 3A,B. The PCA analysis and PLS-DA model of both the male and female date leaf samples were also built using FT-IR/ATR transformed spectra. The results of the PCA analysis and PLS-DA model are depicted in Figure 4A,B. Unit vector normalization, SNV, and S. Golay spectral transformation with 10 smoothing points at a polynomial order of 2 were also used to remove the noise from FT-IR spectral data while building the multivariate models. In the FT-IR/ATR data, we noticed distinctive higher intensities of peaks at wavenumbers from 1410 to 1510 cm^−1^, from 2350 to 2410 cm^−1^, and from 3000 to 3500 cm^−1^. The major difference in male vs. female samples was prominent at wavenumber 1490 cm^−1^ that represents the abundances of glycosides and C–H bends, whereas another major differentiation was observed at wavenumber 2380 cm^−1^, suggesting the presence of carboxylic groups associated with polysaccharides and proteins. The PCA analysis classified male and female date leaf samples into two discriminative classes, as shown in Figure 4A. Correspondingly, the PLS-DA model was built for male and female date palm leaves using FT-IR/ATR spectra, as shown in Figure 4B. The PCA-based score-plot for PLS-DA models is also shown in Figure S6. The loading plots for both PCA and PLS-DA are shown in Figures S7 and S8. Various biological samples and related natural products have been assessed and standardized using FTIR-ATR-based data [37]. The current results also support the idea that this method can be used for different sex-related issues in deciduous plants. The results of the infrared spectroscopy were also supported by spectral data of unknown samples of immature date palm leaves. The result shows a clear discrimination of male vs. female in both PCA and PLS-DA regression analysis of NIRS/FTIR data (Figures S9 and S10). 

### 2.3. NMR Based Validation of Sex Differentiation in Date Palms

**Figure 5:**
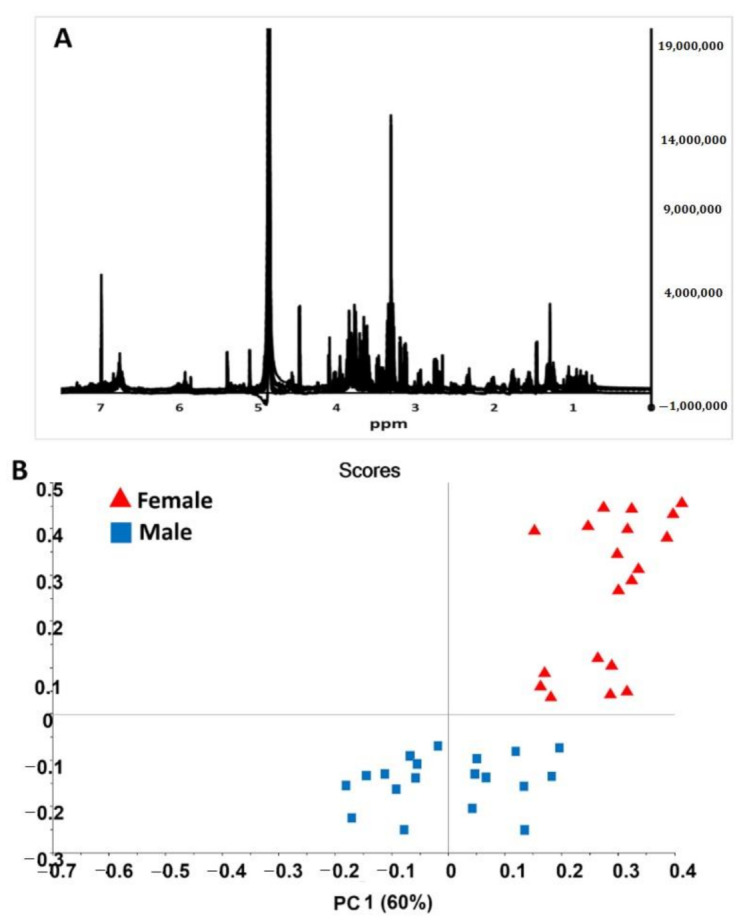
NMR spectral data analysis (**A**) NMR spectra of male and female date palm leaf extract samples from 20 male and 20 female samples. (**B**) PCA score plot of NMR spectral data of male and female date palm leaf extract samples.

The ^1^H-NMR spectra for male and female date palm leaf extract samples are shown in Figure 5. From the spectral data, we found extensive NMR signals in the mid to low frequency range—between δ5.5 and δ3.0 ppm. These signals were characterized as representing glucose/fructose and/or maltose/sucrose. Both of them are di- and monosaccharides. Similarly, during the analysis, less intensive ^1^H-NMR signals were noted in the δ7.0–δ6.0 ppm and δ3.0–δ0.5 ppm regions. However, more complex spectral data were revealed, and this required the use of further multivariate techniques to differentiate between males and females. The PCA of the ^1^H-NMR spectra for sex differentiation is shown in Figure 5B, which clearly illustrates two considerable clusters, representing the two sexes. The representative PC1 vs. PC2 of the male and female leaf samples were mapped and spanned, and this was based on the differences in their respective NMR spectra due to changes in the chemical shifts.

Further demarcation of male and female leaf parts was performed by loading plots of related PCA models on the ^1^H-NMR spectra (Figure 5). This allowed us to identify signaling variables (e.g., in this case, chemical shifts) as the major cause of cluster formation in the ^1^H-NMR spectral data. The interpretation of the NMR signal suggested that primary sugars are the sole reason for differentiation. However, minor resonances also play roles in sex differentiation. A previous study conducted NMR-based profiling of human obesity in individuals of different sexes [40]. This method has been used extensively for human- and animal-related sex discrimination; however, it was used for the first time here to differentiate between sexes in plant samples. 

### 2.4. Molecular Marker Analysis of Sex-Specific Traits in Date Palms

**Figure 6:**
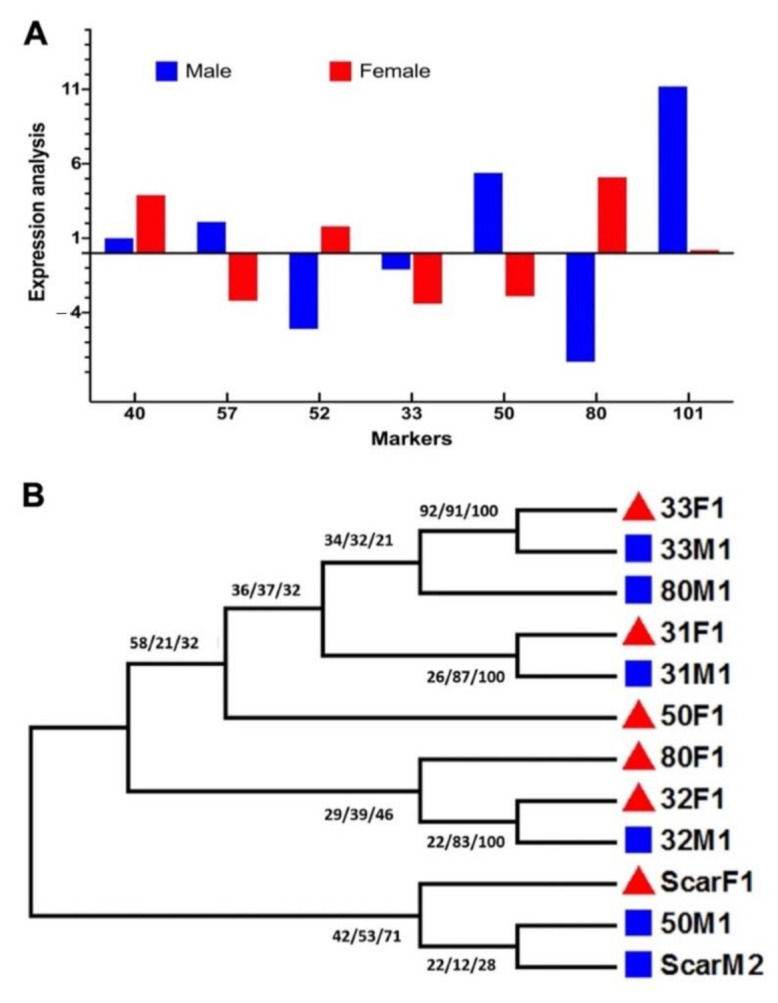
Molecular analysis of markers related sex differentiation in immature leaf samples of date palm. (**A**) Gene expression and transcript accumulation related to sex-linked differentiation in male and female date palms. The up and down bars also represent up or down expression of a specific gene. (**B**) Detailed phylogenetic analysis using maximum parsimony (MP), maximum probability (ML), and neighbor-joining (NJ) methods with 1k bootstrapping and clade formation among male and female samples using specific loci to differentiate between the two sexes in date palms. Bars represent the means ± SE (*n* = 3). MA/FA = actin control; F = female; M = male.

To further validate the results of the reflectance spectroscopy, a detailed molecular marker analysis was performed using sex-specific molecular markers (Table S1). The test for loci with sex-specific alleles and microsatellite markers (Table S1) showed that out of three sex-based loci, only mPdIRDP50 showed clear discrimination of sex between male and female samples. The RT-PCR data showed a detailed transcript accumulation of male samples rather than female samples (Figure 6A). Further qPCR analysis of specific taq-labeled genes showed that there was significantly higher expression of PDK-101, mpdCIR57, and mPdIRDP50, which suggests the broader future application of these genes as specific markers for sex differentiation in date palms. Among these three genes, PDK101 was able to discriminate between sexes the best, followed by mPdIRDP50. mpdCIR57 showed the lowest level of expression in this case. In addition to current findings, in previous reports [15,16,17,18,19,20], mPdIRDP80, mPdCIR078, mPdCIR031, and mPdCIR040 were found to be not fully expressed to allow the differentiation of males, but they showed significantly variable expression for female samples. This suggests that the method could also be used to specifically standardize female samples. mPdIRDP52 and mPdCIR033 failed to be expressed fully in the two samples; this was also shown by the results of the reverse transcription analysis (Figure 6A).

In addition to the RTPCR analysis for transcript accumulation, we used 14 distinct SSRs to assess putatively sex-linked date palm genome scaffolds, and it was observed that they were possibly sex-linked. The six loci demonstrated substantially greater genetic differences between sexes, as was also previously measured with the R*st* index [20]. These six loci that were successfully amplified were mPdIRDP80, mPdIRDP50, mPdIRD033, mPdIRD031, mPdCIR032 [16,20], and SCAR dp [3]. With the specific veriflex annealing temperature, the success rate was 100% for three consecutive PCR replications. The remaining eight loci (Table S1) either did not show any amplification with the samples or were often confronted with PCR dimer contamination. Previously, a set of SSRs identified from the genomic datasets (mPdIRD033, mPdCIR078, and mPdIRD040) exhibited low or no significant R*st* value, which is also in similar to our findings. We found that markers from scaffolds previously segregated by sex were all located in the lower half of LG12, indicating differentiation based on the sex of the chromosome. Notably, male samples were heterozygous for the three loci that showed sex linkages because of male-specific alleles, as recently reported [20].

Further, looking at the genetic differentiation of male and female samples, an extensive PCR analysis was performed, followed by sequencing of the specific products. The results (Table S2) show that based on sequences obtained from six SSR loci, *He* and *Ho* generate autosomal loci that are significantly different between sexes. The *Ho* values for the male group (0.690) and female group (0.482) revealed wide genetic diversity within sexes. The R*st* indexes determine genetic discrimination in the six loci of males, which indicates the presence of structure and is consistent with allele exchange in both sexes. A similar observation with a higher R*st* value was observed by [20], suggesting the reproducibility of the current and previous datasets for sex differentiation in date palms. In addition to date palms, other species such as *Hippophae rhamnoides* ssp. Turkestanica have shown discrimination by gender in analyses using SSR-based molecular markers [41].

Furthermore, in the SSR loci mPdIRD031 and mPdCIR032, a consensual sequence CAC TTG CTT CCT CTG was also revealed after consecutive sequence alignment. However, among all the six SSR markers, no specific homology for nucleotides existed. In terms of the transition and transversion bias (R = 1.022) between the markers, a nucleotide substation rate of –2790.616 existed for 30 sequences of both males and females. Maximum composite likelihood estimates of the pattern of nucleotide substitution suggested that the nucleotide frequencies are 26.72% (A), 25.11% (T/U), 25.37% (C), and 22.80% (G). The transition/transversion rate ratios are k1 = 1.776 (purines) and k2 = 2.312 (pyrimidines). Additionally, Tajima’s neutrality test showed a nucleotide diversity of 0.654 in male compared with female samples, and there were 122 segregating sites. The detailed phylogenetic analysis using the ML, MP, and NJ algorithms with 1k bootstrapping showed a clear distinction of clade formation among male and female samples using specific SSR loci to differentiate between the two sexes in date palms (Figure 6B). The phylogeny showed that the mPdIRD033, mPdIRD031, and mPdCIR032 markers formed a clear clade with more than 70% similarity as compared to other markers. SCAR dpF, mPdCIR050, and mPdCIR080 showed divergence among clades. A report by El-Yazal et al. [19] focused on sex identification in seedlings of date palms using random amplified polymorphic DNA markers; however, more recently, studies have suggested the use of nuclear simple sequence repeat loci as an ideal approach to distinguish among wide varieties of date palm cultivars with a few markers to validate the sex-linked traits [10,16,17,20]. The current study concludes that although some of the sex-linked molecular markers have been reported and developed, there is still a need to develop more robust methods for timely sex differentiation in date palms.

## 3. Materials and Methods

### 3.1. Plant Collection and Sampling

Leaf samples from date palm (*Phoenix dactylifera* L.) were collected from a farm in Birkat Al-Mouz, Nizwa Sultanate of Oman (22°54′23.39′′ N 57°40′11.99′′ E). These samples were from 50 male (fahal) and 50 female (khalas) trees. The environmental and growth conditions and the ages of the trees were similar. Offshoot immature leaf from the male and female trees were collected. Traditionally, after 5 to 8 years, the gender of a date palm tree becomes known as it either produces pollen or fruit, whereas in the early developmental stage, the gender is generally unknown unless the parent of the shoot is known. This the reason why we also collected samples with known and unknown parents to ensure cross-validation of the method. To maintain the integrity of the collected leaf samples, they were brought to the Lab on liquid nitrogen, ground to a fine powder, and shifted immediately to –80 °C for (i) IR, (ii) NMR, and (iii) molecular analyses. 

### 3.2. Spectral Analysis using NIRS, FTIR, and NMR

The method described by Rehman et al. [27] was adopted for this work with some modifications. Briefly, the powdered leaf samples were extracted with methanol (MeOH: water—80:20 LC grade) three times to form an extract. We used three spectroscopic analysis methods: (i) near-infrared reflectance-spectroscopy using wavelengths from 10,000 to 4000 cm^−1^, (ii) Fourier-transform infrared attenuated-total-reflectance spectroscopy (FTIR/ATR) in the wavelength range of 400 to 4000 cm^−1^, and (iii) nuclear magnetic resonance molecular absorption spectroscopy. The reflectance-NIRS of both male and female date palm leaf samples was recorded using NIR (BSEN60825-1:2007; Perkin Elmer, Boston, MA, USA) with trans-reflectance accessories, providing a 0.5 mm pathlength. For FTIR-ATR, the powdered leaf samples from the male and female samples were read separately five times to create a consensus spectrum. Similarly, the mid-infrared spectra of both the male and female date palm leaf samples were recorded with an FT/IR Spectrometer (Tensor 37; Bruker, Hamburg, Germany). The number of scans conducted for each leaf sample was 32, and there were done at a resolution of 4 cm^−1^ in reflectance mode.

The immature leaf samples were also analyzed with ^1^H-NMR where each sample was prepared in 550 µL of methyl-d_3_ alcohol-d. All ^1^H-NMR measurements were performed with the Bruker-Advance III HD 600MHz spectrometer (Bruker Biospin, Fallanden, Switzerland) using a 5-mm double resonance broadband probe with a Z-gradient coil equipped with a Bruker Automatic Sample Case of 24 samples. The ^1^H-NMR spectra were acquired using 1D sequencing with a 30-degree flip angle at 298.2 K without sample rotation. Thirty-two scans and two prior dummy scans of 32 K points were taken at a spectral width of 20.0269 ppm, a receiver gain of 114, and an acquisition time of 1.363 s, with each sample requiring about 1 min 55 s for analysis. Spectral data were processed using Bruker Topspin software 3.2 (Hamburg, Germany). Prior to the Fourier transformation, the FIDs were multiplied by an exponential weighting function, leading to a line extension of 0.30 Hz. The individual parts of the transformed spectra were translated to ASCII files in XY plot format. Each ASCII file was modified to delete the header text and X-data and then read in as a column into an MS EXCEL spreadsheet.

### 3.3. Multivariate Data Analysis

Multivariate chemometric models, like the principal component analysis (PCA) and PLS-DA, were used to assess the spectral data to investigate the first level of sex discrimination in date palm leaf samples. Furthermore, a revalidation approach was adopted to identify the basis of known and unknown leaf samples of date palm. Unscrambler (version 9.0; Gaustadalléen, Norway) and MS Excel (version 2010) tools were used. The PCA study used random cross-validation, while the internal validation methods used the PLS-DA (leave-one-out cross-validation model). Various methods used for transforming spectra, such as unit-vector normalization, the multiplicative scattering method (MSC), standard normal variate (SNV), baseline correction, and 1st derivative functions at a polynomial order of 2 with Savitzky–Golay including 7 smoothing points, were applied to the spectral data, and noise was removed.

### 3.4. PCR, RT-PCR, and qPCR-Based Analyses of Selected Genes/Molecular Markers

Date palm leaf samples, collected from trees were transferred into a −80 °C freezer, and a modified DNA extraction protocol with cetyl trimethyl ammonium bromide (CTAB) was used, according to the method presented by Cherif et al. [20]. The precipitated genomic DNA was collected and each pellet was dried before being re-dissolved in 100 μL of TE buffer (10 mM Tris-HCl, 1 mM EDTA). The DNA yield was calculated by a Qubit 3.0 Fluorometer (Thermo Fisher Scientific, Q33217, Gloucester, UK). The data obtained were used to analyze date palm loci that have been identified to differentiate sex (Cherif et al. [20] and Al-Mahmoud et al. [15]) whilst using modified PCR conditions. Moreover, RNA was extracted from the samples of the leaves using a modified version of the protocol presented by Chan et al. [26]. A 2× real-time PCR kit (BioFACT, Seoul, South Korea), 10 nM of each gene-specific primer, and 100 ng of template cDNA were used in 20 μL reaction mixtures, and the whole reaction was performed in compliance with the standard manufacturer’s protocol using Quant Studio 5 (Applied Bioscience, CA, USA) as a negative guide. Gene expression was associated with actin expression as an internal regulation method, and each test was replicated three times.

### 3.5. Genetic Analysis

PCR products were analyzed using Genetic Analyzer (ABI3130XL; Macrogen Inc. Korea; Applied BioSystems, Foster City, CA, USA). The size of each allele was scored using GeneMapper v3.7 (Applied BioSystems, CA, USA) software, as proposed by Cherif et al. [20] and Peakall and Smouse [27]. We contrasted the observed frequencies of heterozygotes (*Ho*) for male and female groups with those predicted to presume genotype frequencies of Hardy–Weinberg (*He*) using the GenAlEx 6.41 program [42]. Four approaches, including Bayesian inference (BI) introduced with MrBayes 3.1.299, maximum parsimony (MP) with PAUP 4.0100, maximum probability (ML), and neighbor-joining (NJ) with MEGA 7.0 were used to build the phylogenetic trees. Using the Kimura 2-parameter model with gamma-distributed variable heterogeneity and invariant sites, parameters for the ML analysis were designed with a BIONJ tree used as the founding tree with 1000 bootstrap replicates.

### 3.6. Statistical Analysis

Unscrambler version 9.0 was used for statistical research, as described by Rehman et al. [27]. Multivariate approaches for exploratory data processing, such as PCA and PLS-DA, were extended for use with spectral data. The PCA analysis helped us to examine the similarities as well as the diversity between male and female samples. The most strongly related spectral regions of the database and spectral pre-processes of the plant samples were specified. To remove the noise from the spectral data, different types of spectral pre-processes, such as unit vector normalization, multiplicative scatter correction (MSC), standard normal variate (SNV), and 1st derivative Savitzky–Golay, were applied. Random cross-validation was conducted as an internal validation technique by implementing the leave-one-out cross-validation procedure of the PLS-DA model. To assess the significance of gene expression, an analysis of variance (ANOVA) with multiple comparisons was used (*p* < 0.05). Multiple Student t-tests were also administered using the Sidak–Bonferroni procedure (*p* < 0.05) to calculate statistical significance and statistically evaluate the mean values and standard deviations. In addition, graphical interpretation was conducted using GraphPad Prism v6.01. (GraphPad Software Inc., San Diego, CA, USA).

## 4. Conclusions

The current study elucidates the use of reliable and authentic spectroscopic methods with the help of chemo-informatics regression analysis to discriminate among samples. Numerous reports have suggested the use of molecular markers to differentiate between male and female date palm cultivars, e.g., Bekheet and Hanafy [14], Younis et al. [11], Al-Dous et al. [12], Al-Mahmoud et al. [15], Elmeer and Mattat [16], Zhao et al. [17], and Cherif et al. [20]; however, the discriminative percentage can vary greatly. Additionally, molecular methods are more sensitive and require gentle handling, and are expensive too. In contrast, in the current study, the PCA model based on NIRS and FTIR/ATR (followed by NMR validation) was able to differentiate between male and female samples, suggesting the legitimacy of the method. Utilizing such methods could help tissue culture experts and breeders involved in establishing explant stocks of various cultivars.

## Data Availability

The data presented in this study are contained within the article and supplementary material.

## Supplementary Materials

The following are available online at https://www.mdpi.com/2223-7747/10/3/536/s1, Figure S1: NIR spectra (without pre-processing) of male (A) and female immature date palm leaf samples, Figure S2: PCA model loading plot of the NIR spectral data of both male and female immature date palm leaf samples, Figure S3: PLS-DA score plot using the NIR spectral data of male and female date palm leaf extract samples, Figure S4: PLS-DA loading plot using the NIR spectral data of male and female date palm leaf extract samples, Figure S5: PLS-DA loading plot using the NIR spectral data of male and female date palm leaf extract samples, Figure S6: PCA Loading plot using FTIR ATR spectral data of male and female date palm leaf samples, Figure S7: PCA plot for PLS-DA using FTIR ATR spectral data of male and female date palm leaf samples, Figure S8: PLS-DA loading plot using FTIR ATR spectral data of male and female date palm leaf samples, Figure S9: PCA model loading plot of the NMR spectral data of both male and female date palm leaf extract samples, Figure S10: NIRS of unknown samples at immature stage of date palm, Figure S11: PLS-DA plot using NIRS spectral data for unknown samples at immature stage of date palm, Table S1: Primers used for identifying the gender specific traits in date palm samples, Table S2: Estimated allele frequencies and estimated heterozygosity and other analyses for all populations.
