# Supplementary material for: Spectroscopic and Molecular Methods to Differentiate Gender in Immature Date Palm (Phoenix dactylifera L.)
Source: Plants (Basel). 2021 Mar 12;10(3):536. doi: 10.3390/plants10030536 (PMC8001243; doi:10.3390/plants10030536)
Supplement: Supplementary file 1 [file plants-10-00536-s001.pdf]

plants

MDPI

Supplementary material

Spectroscopic and molecular methods to differentiate gender in immature date palm (*Phoenix dactylifera* L.)

Abdul Latif Khan [1,†], Ahmed Al-Harrasi [1,†,*], Muhammad Numan [1], Noor Mazin AbdulKareem [1], Fazal Mabood [2,*] and Ahmed Al-Rawahi [1]

[1] Chair of Oman's Medicinal Plants & Marine Natural Products, University of Nizwa, Nizwa, Oman
[2] Department of Chemistry, University of Swat, Khyber Pakhtunkhwa, Pakistan
* Correspondence: aharrasi@unizwa.edu.om (A.A.H.); fazal@uswat.edu.pk (F.M.)
[†] equally contributed.

Supplementary Tables

Table S1. Primers used for identifying the gender specific traits in date palm samples.

SSR Locus	GeneBank ID	Primer Sequences	Tm (°C)
mPdIRDP50	PDK_30s1202771	F:CATGGAAGTTGTTGGCAGAG<br>R:CATGCTCCTTGCCTCAATG	60
mPdIRDP52	PDK_30s680001	F:TCGTGCTACAATGCCAAGAG<br>R:CTAATGCTTGCATGGGAGGT	60
mPdIRDP80	PDK_30s6550963	F:ATTGGGTGTTGGTCTCTAGGAA<br>R: TCGTGCTACTGCTTCTCCATTA	60
mPdCIR078	AJ571685	F:TGGATTTCCATTGTGAG<br>R:CCCGAAGAGACGCTATT	50
mPdIRD031	PDK_30s801751	F:GCAGGTGGACTGCAAAATCT<br>R:CTATTGGGGTGCTGATCCAT	60
mPdIRD033	PDK_30s712151	F:GGAGCATACAGTGGGTTTGC<br>R:CAGCCTGGGAATGAGGATAG	60
mPdIRD040	PDK_30s862741	F:GAGAGATGCGTCAGGGAATC<br>R:CCAGAATCTTCCAAGCAAGC	60
PDK-511		F: GCCACCACATCGAAGTCATCAACGA<br>R: TTGGGACAACCCTTGCCATATG	
PDK-101		F: CCATGCTCAATGCCCTTAGTCGCCTACCGG<br>R: TTGACTTTATCGAAGGGTTACCGTTG	
PDK-131		F: GAGGTACTTCTCAAAGAATCTGTAGA<br>R:TTCAACATTTTGGGTCAGGTCTAGGT	

Loci belonging to scaffolds linked to gender: mPdIRDP50, mPdIRDP52, mPdIRDP80; diversity microsatellite loci: mPdCIR078, mPdCIR031, mPdCIR033, mPdCIR040.

Table S2. Estimated Allele Frequencies and Estimated Heterozygosity and other analysis for all Populations.

Gender	*n*	A	*Ne*	*Ho*	*He*	*p-Value*	*Rst*	% variation
Male	20	8	6	0.690	0.53	0.016	0.534	81.67
Female	20	5	3	0.482	0.31	0.34	0.108	12.394

n = no. of samples, Na = No. of Different Alleles, Ne = No. of Effective Alleles, I = Shannon's Information Index, He = Expected Heterozygosity, uHe = Unbiased Expected Heterozygosity.

Supplementary figures

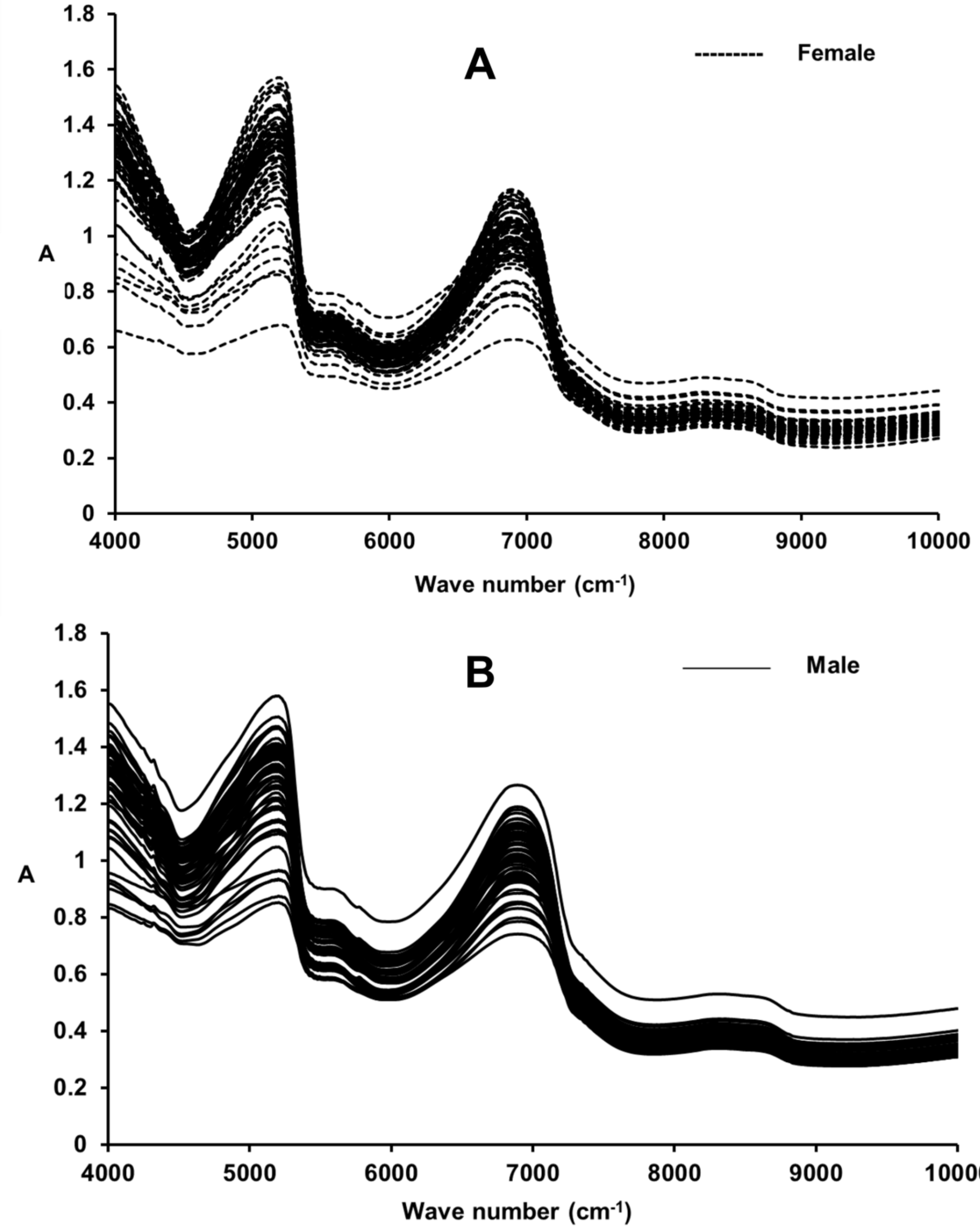

Figure S1. NIR spectra (without pre-processing) of male (**A**) and female immature date palm leaf samples (**B**). A scattering effect was observed due to absorbance without preprocessing in the wavelength range from 4000 to 10,000 cm^{-1}. The spectra represent the fifty individual samples that were tested five times.

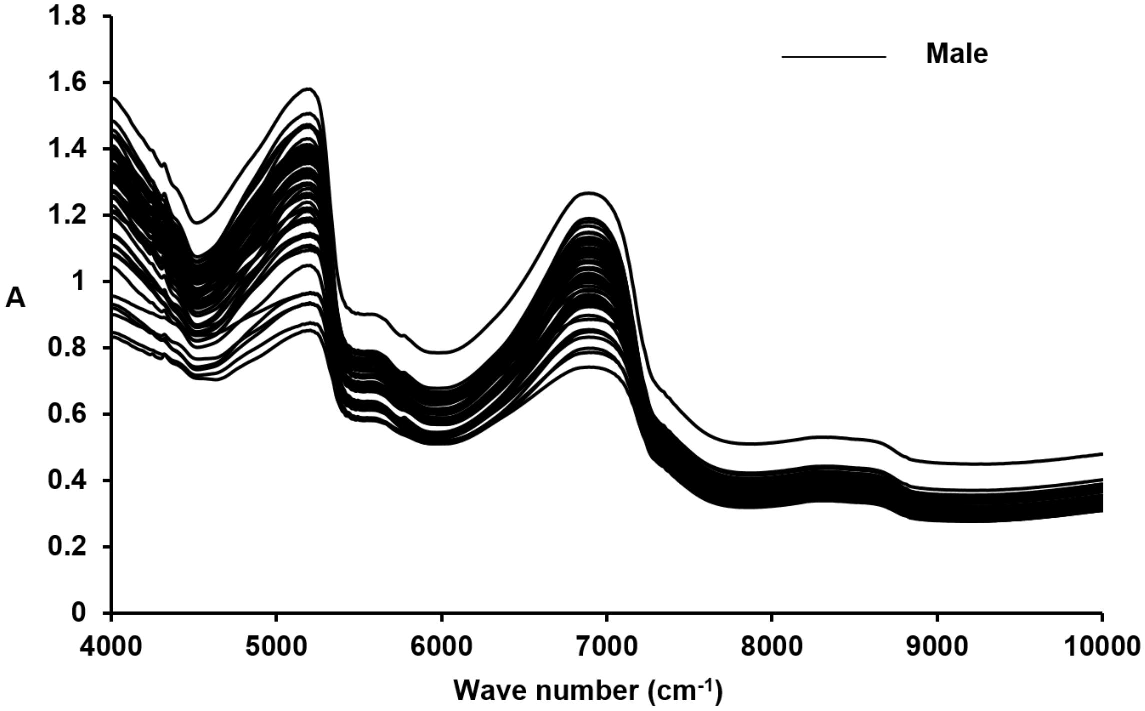

Figure S2. PCA model loading plot of the NIR spectral data of both male and female immature date palm leaf samples.

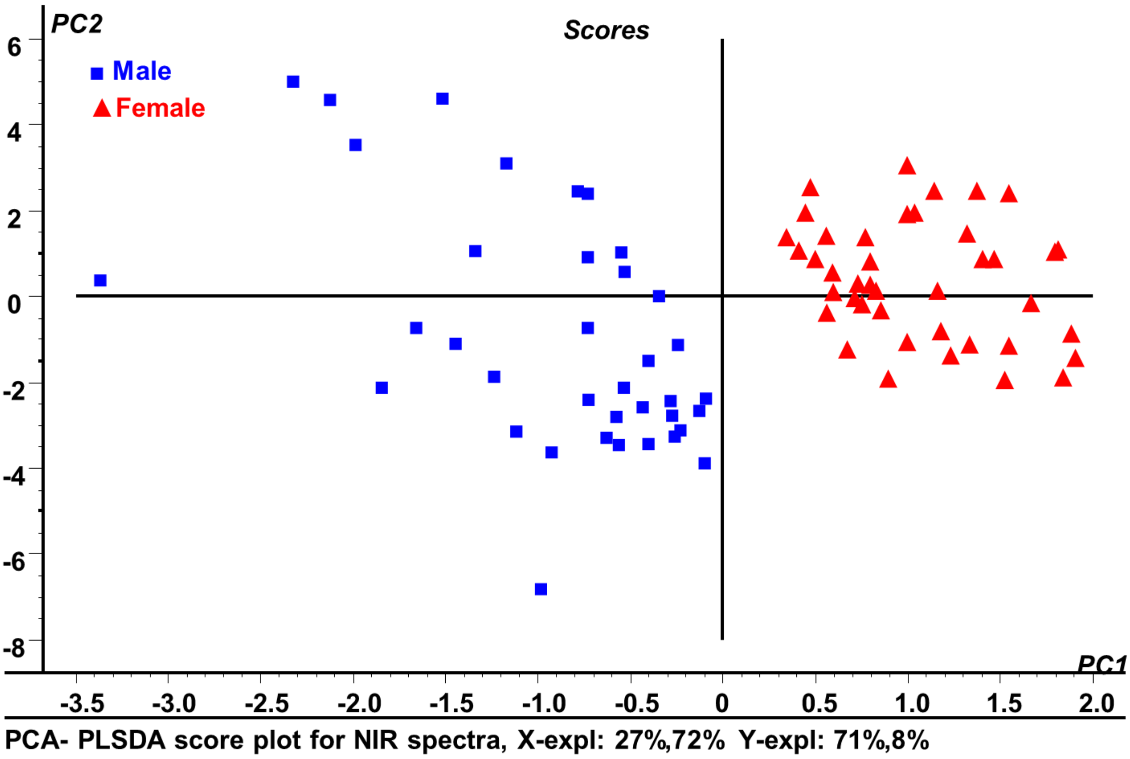

Figure S3. PLS-DA score plot using the NIR spectral data of male and female date palm leaf extract samples.

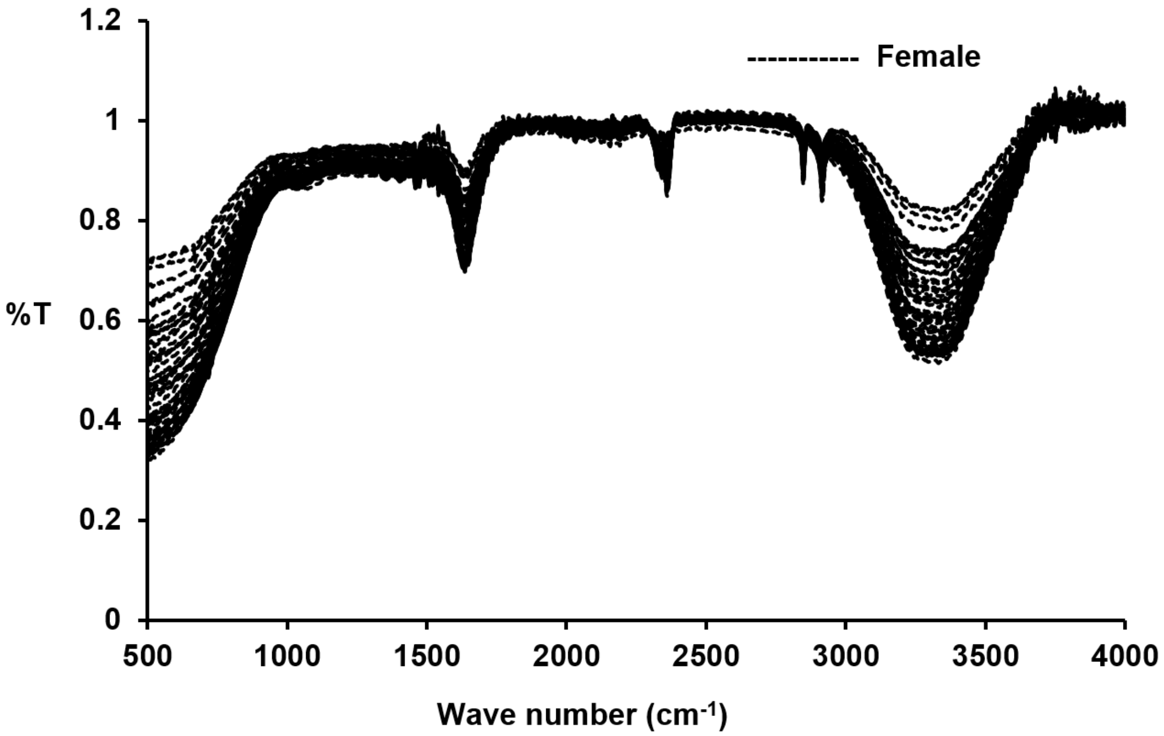

Figure S4. PLS-DA loading plot using the NIR spectral data of male and female date palm leaf extract samples.

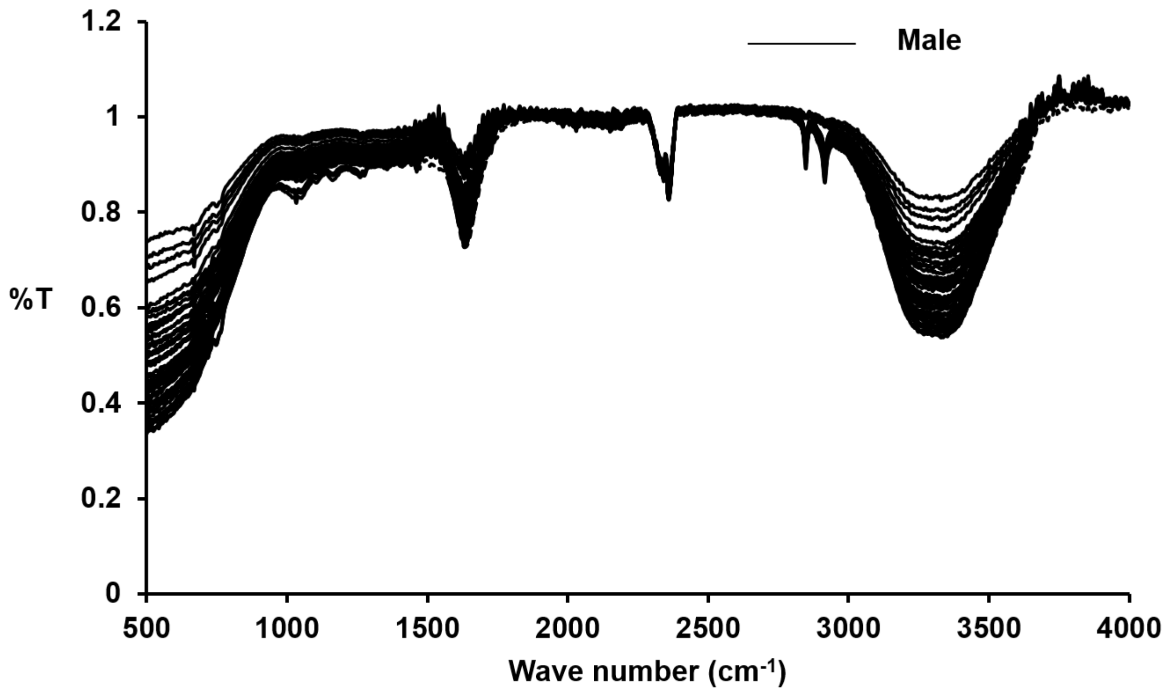

Figure S5. PLS-DA loading plot using the NIR spectral data of male and female date palm leaf extract samples. The scattering effect was observed due to absorbance without preprocessing in the wavelength range of 4000 to 10,000 cm^{-1}. The spectra represent the fifty individual samples that were tested five times.

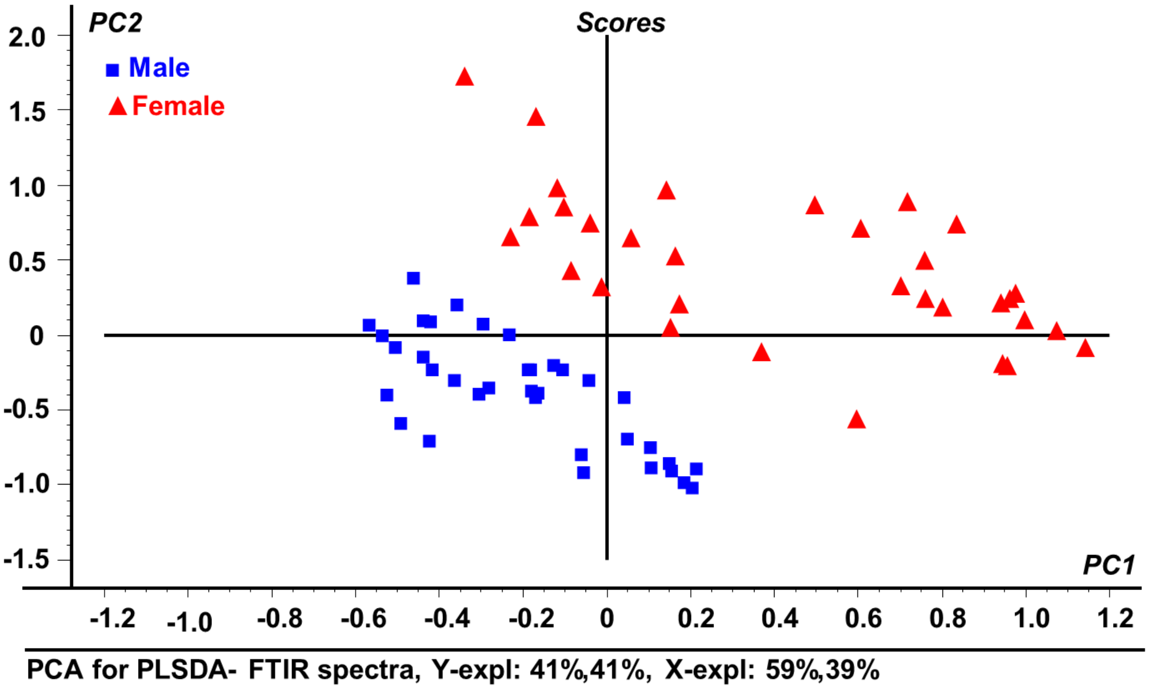

Figure S6. PCA Loading plot using FTIR ATR spectral data of male and female date palm leaf samples.

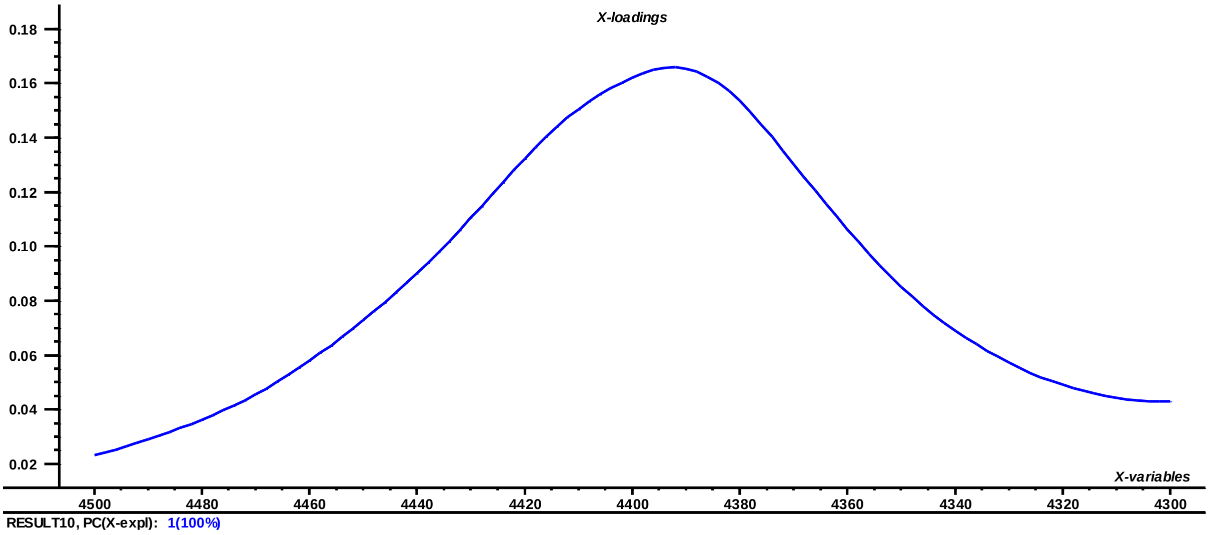

Figure S7. PCA plot for PLS-DA using FTIR ATR spectral data of male and female date palm leaf samples.

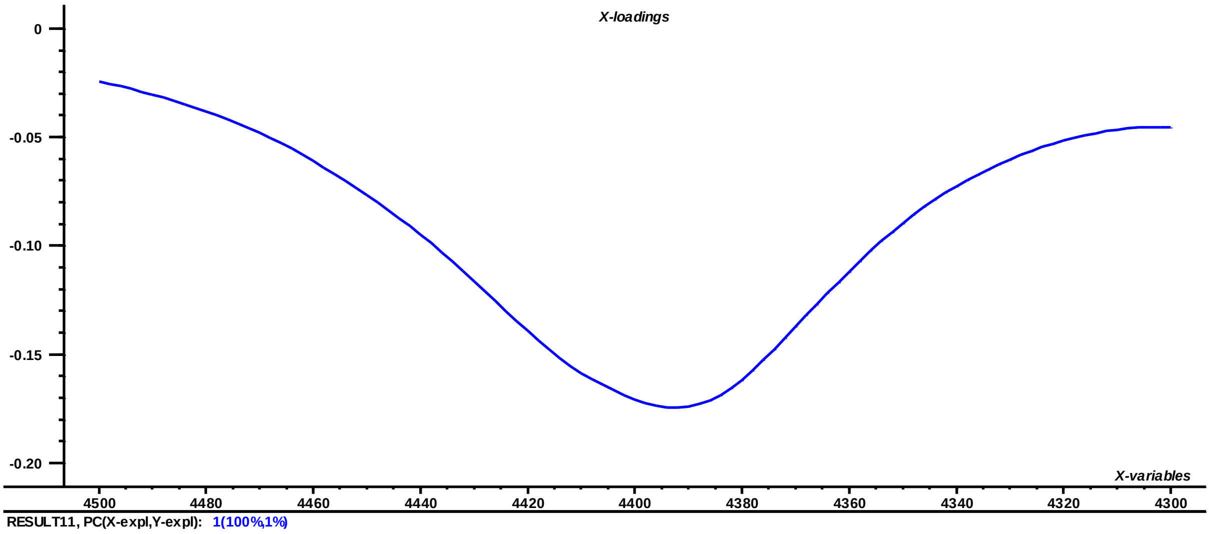

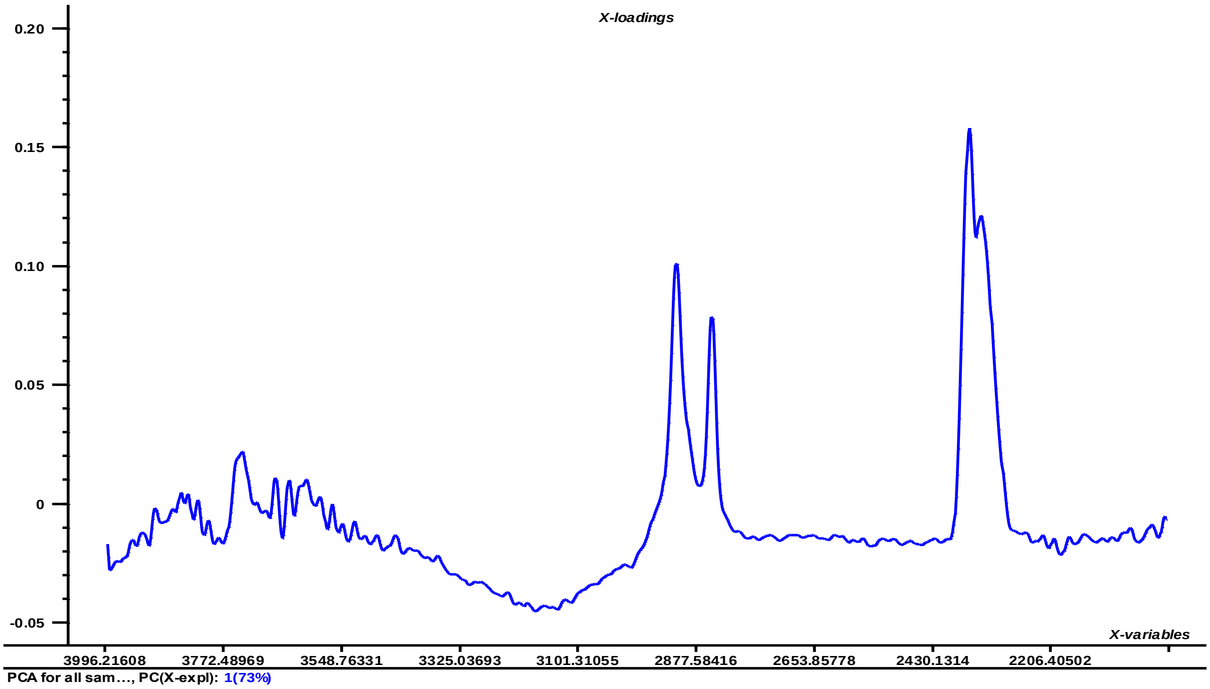

Figure S8. PLS-DA loading plot using FTIR ATR spectral data of male and female date palm leaf samples.

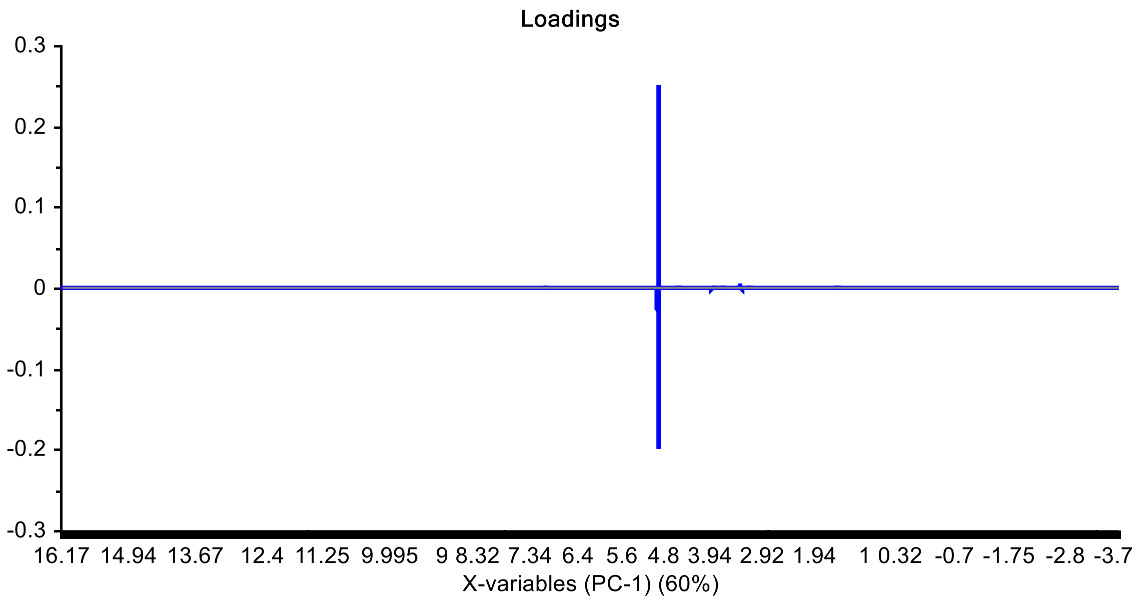

Figure S9. PCA model loading plot of the NMR spectral data of both male and female date palm leaf extract samples.

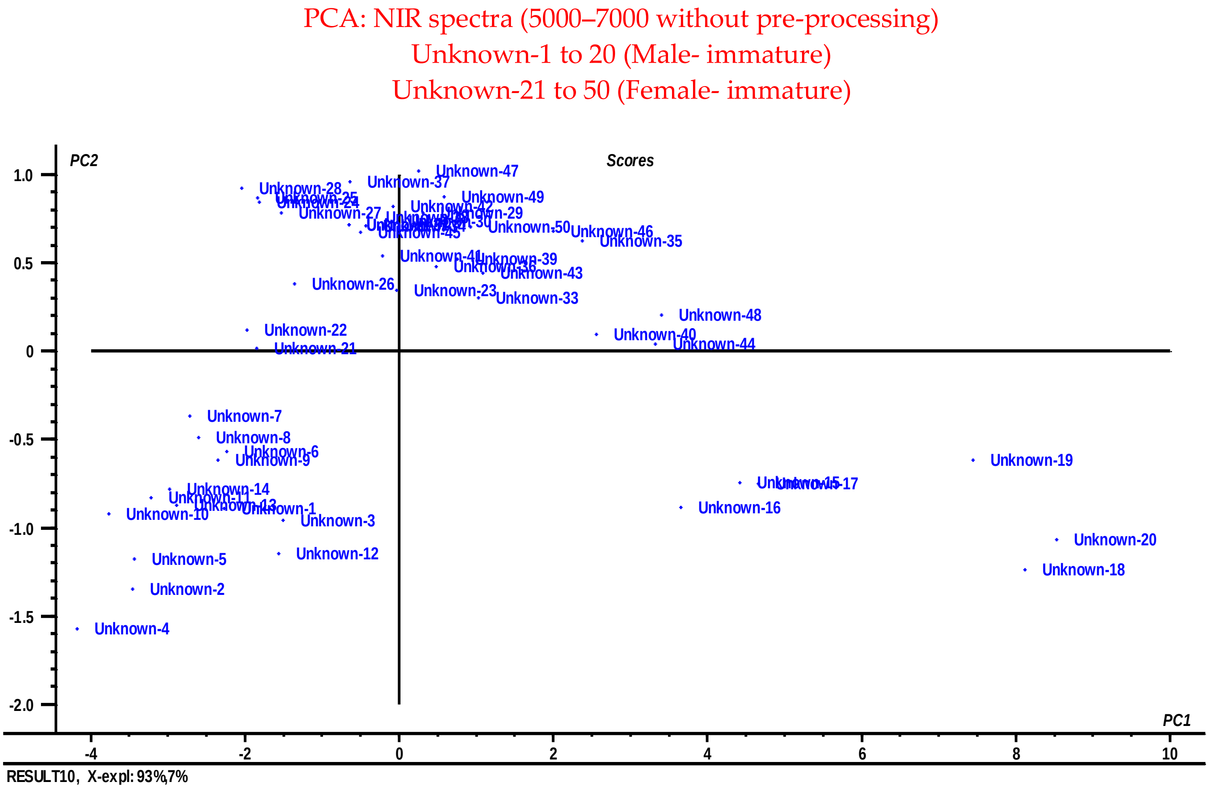

Figure S10. NIRS of unknown samples at immature stage of date palm.

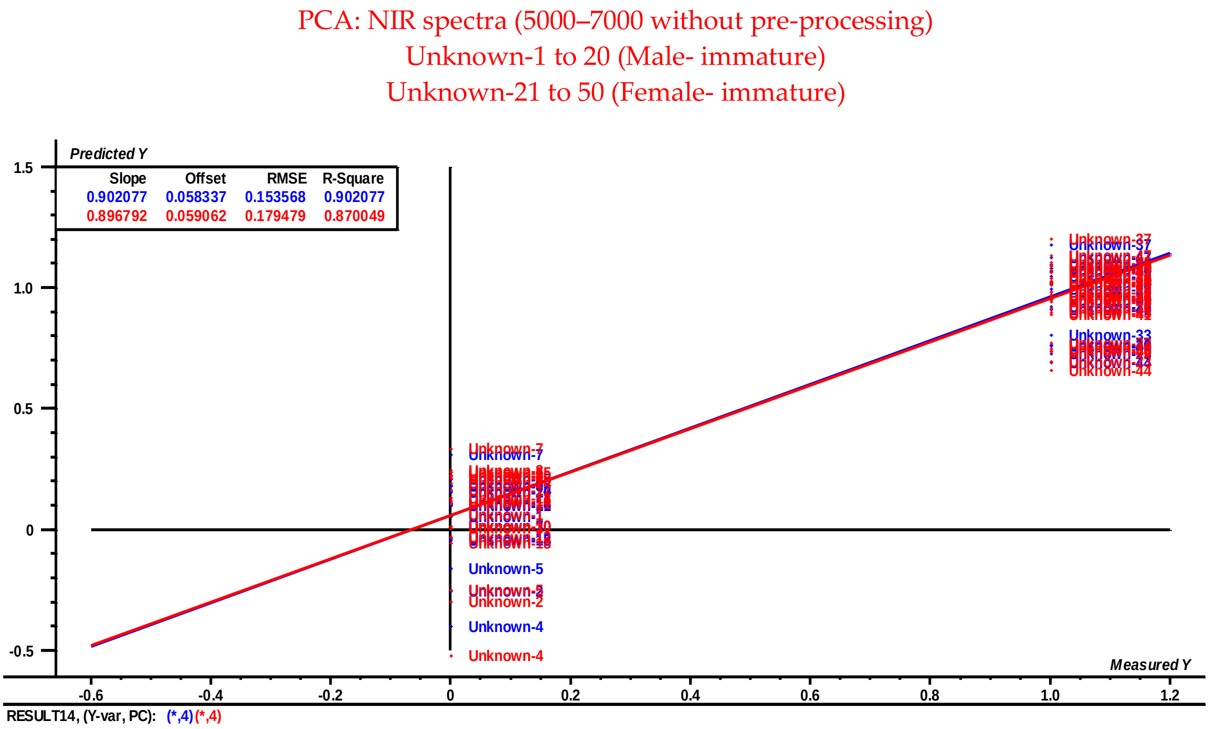

Figure S11. PLS-DA plot using NIRS spectral data for unknown samples at immature stage of date palm.
